# Association between oral microbiome diversity and kidney stones: a cross-sectional study

**DOI:** 10.3389/fmicb.2025.1600961

**Published:** 2025-05-29

**Authors:** Zufa Zhang, Sheng Guan, Li Chen, Fengze Jiang, Huqiang Dong, Zuyi Chen, Long Lv, Hongxuan Song, Weibing Sun, Danni He, Sixiong Jiang, Feng Tian

**Affiliations:** ^1^Affiliated Zhongshan Hospital of Dalian University, Dalian, Liaoning, China; ^2^Zhongshan Clinical College of Dalian University, Dalian, Liaoning, China; ^3^Key Laboratory of Microenvironment Regulation and Immunotherapy of Urinary Tumors of Liaoning Province, Dalian, Liaoning, China; ^4^Fuzhou Children's Hospital, Fujian Medical University, Fuzhou, Fujian, China; ^5^School of Public Health, Ningxia Medical University, Yinchuan, Ningxia, China

**Keywords:** oral microbiome diversity, kidney stones, National Health and Nutrition Examination Survey (NHANES), observed ASVs, faith's phylogenetic diversity, Shannon-Weiner index, Simpson Index, alpha diversity

## Abstract

**Background:**

Kidney stones are a common urologic disorder that imposes a significant burden on global public health. This study aimed to determine the association between oral microbiome diversity and kidney stones.

**Methods:**

The data for this study came from the National Health and Nutrition Examination Survey 2009–2012 survey cycle. Use of alpha diversity to assess oral microbiome diversity. Multivariate logistic regression modeling was used to assess the association between different alpha-diversity indicators and kidney stones. Subgroup analyses and interaction tests were used to assess the stability of the association between alpha-diversity and kidney stones. Restricted cubic spline plots were used to assess non-linear associations and dose-response relationships.

**Results:**

The study included 5,870 eligible participants with a mean age of 43.74 years at baseline. After adjusting for all covariates, the observed oral microbiome diversity was significantly negatively associated with the risk of kidney stones (*P* < 0.05). Subgroup analyses showed that oral microbiome diversity was negatively associated with the risk of kidney stones in certain populations, particularly among those aged 40–60 years, men, obese, with moderate to high cardiovascular health scores, smokers, and those without hypertension. Restricted cubic spline analysis suggested a significant non-linear negative correlation between the Shannon and Simpson diversity indices and the risk of kidney stones (*p* for non-linear < 0.05). Since our study was a cross-sectional design, the main limitation was the inability to prove causality.

**Conclusions:**

In this study, we found an inverse relationship between oral microbiome diversity and kidney stone risk observed in alpha diversity. This reveals the complexity of host-microbiome interactions, and further mechanistic studies are necessary to elucidate these complex roles in the future.

## 1 Introduction

Kidney stones are a common urologic disorder that imposes a significant burden on global public health (Thongprayoon et al., 2020). The global incidence of kidney stones has increased in recent decades, particularly in Western countries and several emerging economies (Romero et al., 2010). In recent years, the diagnosis of kidney stones has gradually increased, driven by changes in lifestyle, dietary habits, and advancements in medical technology (Dai et al., 2019; Ferraro et al., 2020). Studies indicate that ~50% of individuals with kidney stones will experience recurrence within 5–10 years of the initial episode (Siener, 2021). For patients with kidney stones, taking appropriate preventive measures can effectively reduce the probability of recurrence (Fontenelle and Sarti, 2019). Understanding the epidemiologic characteristics of kidney stones and their risk factors is crucial for developing effective prevention and treatment strategies, ultimately reducing the public health burden.

Oral microbiome diversity refers to the variety and abundance of microorganisms, including 56 bacteria, fungi, and viruses, in the oral cavity (Berg et al., 2020). Among the various microbial habitats in the human body, the oral microbiota stands out as one of the most intricate and densely colonized communities (Baker et al., 2024). The diversity of the oral microbiome is essential not only for maintaining oral health, but it also has a significant association with overall systemic wellbeing (Graves et al., 2019). Increasing evidence suggests that an imbalanced oral microbiota may not only lead to oral diseases but also be linked to systemic conditions such as cardiovascular disease, diabetes, and respiratory infections (Wang et al., 2024; Hosomi et al., 2022; Crestez et al., 2024). However, there are fewer direct studies on the oral microbiota and kidney stones, but there are some studies in related fields that provide insights. Research has demonstrated that dysbiosis of the oral microbiota is associated with the advancement of chronic kidney disease, particularly among individuals suffering from periodontitis (Yasuno et al., 2024). Patients with infectious urinary tract stones frequently experience oral health issues, particularly among the elderly and immunocompromised individuals. An imbalance in the oral microbiota may exacerbate urinary tract infections by increasing pathogenic bacteria in the mouth (Jones-Freeman et al., 2021).

Although direct studies on the oral microbiota and kidney stones are limited, existing evidence indicates that a healthy oral microbiome plays a key role in the host's overall health (Wade, 2013). Population-based studies are essential to explore the relationship between oral microbiota and kidney stones, aiming to promote human health and elucidate the underlying mechanisms. Accordingly, we utilized data from the 2009–2011 and 2011–2012 cycles of the National Health and Nutrition Examination Survey (NHANES), a large-scale population-based dataset, to examine the relationship between oral microbiome diversity and the occurrence of kidney stones.

## 2 Methods

### 2.1 Study population

**Figure 1:**
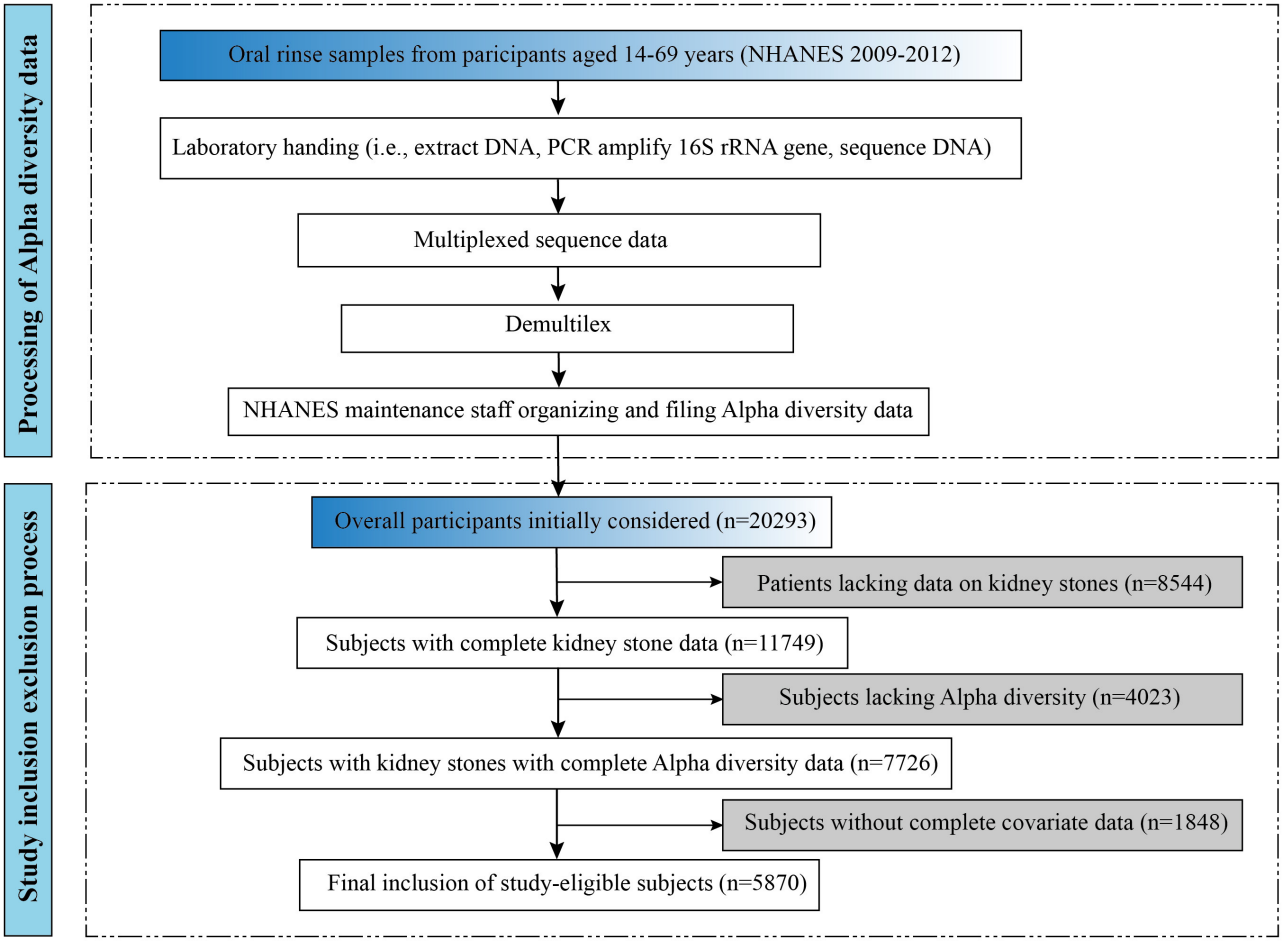
The processing flow of Alpha-diversity and overall study inclusion-exclusion flowchart.

The detailed process of participant inclusion and exclusion for this study is illustrated in the lower portion of Figure 1. This study initially included 20,293 subjects aged 20 years and older. In addition, 4,023 subjects without alpha diversity data were excluded, and on this basis, 1,848 subjects without complete covariates were excluded. A total of 5,870 eligible adult subjects were recruited for this study. Of these, 5,409 individuals with kidney stones and 461 individuals without kidney stones participated in being included in the analysis. The data for this study came from the NHANES 2009–2012 survey cycle, which can be found on the official website at https://www.cdc.gov/nchs/nhanes/.

### 2.2 Bioinformatics and oral microbiome diversity

The processing flow for alpha diversity is shown in the upper part of Figure 1. DNA extraction from oral rinse samples was performed following previously established protocols, as detailed in the NHANES laboratory procedures manual (https://wwwn.cdc.gov/nchs/data/nhanes/2009-2010/manuals/HPV.pdf) (Gillison et al., 2012). Polymerase chain reaction (PCR) amplification and sequencing were conducted in accordance with previously published methods (Caporaso et al., 2012), and further technical details are available on the Earth Microbiome Project website (https://earthmicrobiome.org/protocols-and-standards/16s/). Information about the oral microbiome data file is available on the web page (https://wwwn.cdc.gov/Nchs/Nhanes/Omp/Default.aspx).

Oral microbiome diversity was assessed using alpha diversity, which typically reflects community richness and/or evenness. Measures of alpha-diversity include observed ASV, the Shannon-Weiner index, Faith's PD (PD), and the Simpson index, which are generated based on the rarefaction at 10,000 reads per sample, and we analyzed this saturated rarity. Observed ASVs quantify the total number of ASVs in a locality, while Faith's PD uses phylogenetic tree information to assess richness. The Shannon-Wiener and Simpson indices are used to assess both the richness and evenness of a microbial community.

### 2.3 Outcome variable

Kidney stones were the outcome variable in this study. Trained interviewers employed a computer-assisted personal interview method to inquire whether participants had a history of kidney stones. The renal condition urology questionnaire “Have you ever had kidney stones” was used to calculate the prevalence of kidney stones. Respondents who answered “yes” were defined as having kidney stones.

### 2.4 Covariates

We screened covariates for inclusion in multifactor logistic regression models based on previously reported literature and clinical correlations (Wang et al., 2025; Sun et al., 2025). To account for potential confounding variables, the analysis incorporated several covariates, including age, gender, race and ethnicity, education level, marital status, body mass index (BMI), poverty-income ratio (PIR), cardiovascular health (CVH), smoking status, alcohol use, as well as the presence of diabetes and hypertension. Education levels were categorized as less than high school, high school or GED, above high school. BMI was classified into three categories: < 25 kg/m^2^ as normal weight, 25–30 kg/m^2^ as overweight, and ≥30 kg/m^2^ as obese. PIR was divided into three categories: < 1, 1–3, and ≥3. “Have you smoked at least 100 cigarettes in your entire life?” If you answered yes, you were defined as a smoker. “Drinking alcohol at least 12 times a year?” If you answered yes, you were defined as a drinker. Marital status was further divided into four categories: married, never married, living with a partner, and other. Those who answered “yes” and “borderline” to the question, “Have you ever been told by a doctor that you have diabetes, except during pregnancy?” were categorized as patients with a history of diabetes. Individuals who answered “yes” to the question, “Has your doctor told you that you have high blood pressure?” were categorized as hypertensive. Cardiovascular health was assessed using Life's Essential 8 as recommended by The American Heart Association (AHA). Detailed information for calculating LE8 scores based on NHANES is shown in Supplementary Table S1.

### 2.5 Statistical analysis

According to the guidelines for data analysis on the official NHANES website, we considered the weights, which are in line with the recommendations of the CDC. Continuous variables are presented as mean ± standard deviation (SD), while categorical variables are expressed as percentages. The principal component analysis determined the significance of the different alpha-diversity metrics. The diversity of the oral microbiome, as determined by the observed ASVs, was described at baseline. Multivariate logistic regression modeling was used to assess the association between different alpha-diversity indicators and kidney stones. Multiple covariance screening was used to assess for covariance between covariates and to ensure model stability. Subgroup analyses and interaction tests were conducted to evaluate the robustness of the relationship between alpha-diversity and the presence of kidney stones. Restricted cubic spline plots were used to assess non-linear associations and dose-response relationships. We used the 10%, 50%, and 90% tertiles based on the exposure variable as the nodes for the restricted triple spline, and the exact values are shown in Supplementary Table S2. Spline terms were subsequently added to the logistic regression model to provide flexibility in assessing non-linear relationships. In addition, the log-likelihood ratio test from the saturated threshold effects analysis was used to compare the goodness-of-fit of the linear and non-linear models to support the presence of inflection points. Statistical analyses were conducted using Empower software (X&Y Solutions, Inc., Boston, MA, USA) and R4.4.1 (The R Foundation, http://www.R-project.org). The analysis process retains 4 decimal places to get the exact result. Statistical significance was determined by a two-sided *P*-value of < 0.05.

## 3 Results

### 3.1 Baseline characteristics

We show the baseline situation of this study based on the tertile intervals of the observed ASVs. The study included 5,870 eligible participants with a mean age of 43.74 years at baseline. Of these, 48.76% were female and 51.24% were male. The baseline characteristics of the participants are detailed in Table 1. Participants with high oral microbiome diversity were more likely to be young, male, less educated, not hypertensive, unmarried or living alone, and habitual smokers than those with low oral microbiome diversity. The Pearson correlation coefficient ranged from 0.41 to 0.96 between different alpha-diversity metrics (Supplementary Table S3). There is a strong correlation between the different diversity metrics and they are consistent in measuring the biodiversity of the samples (Supplementary Figure S1).

**Table 1:** Baseline characteristics of the study population by oral microbiome diversity.

		**Oral microbiome diversity**			
**Characteristics**	**Overall (*****n*** = **5,870)**	**Tertile 1**	**Tertile 2**	**Tertile 3**	***P*** **value**
Age (year)	43.74 ± 14.36	47.39 ± 14.34	43.32 ± 14.33	40.50 ± 13.57	< 0.0001
Gender (%)					< 0.0001
Male	3,008 (51.24)	45.75 (42.97, 48.55)	51.86 (48.96, 54.75)	57.91 (54.91, 60.86)	
Female	28.62 (48.76)	54.25 (51.45, 57.03)	48.14 (45.25, 51.04)	42.09 (39.14, 45.09)	
Race and ethnicity (%)					< 0.0001
Mexican American	946 (16.12)	4.90 (3.14, 7.57)	7.46 (5.12, 10.74)	14.86 (10.12, 21.29)	
Other Hispanic	574 (9.78)	3.72 (2.49, 5.52)	5.62 (3.79, 8.25)	7.20 (5.13, 10.03)	
Non-Hispanic White	2,434 (41.47)	76.68 (71.77, 80.95)	69.00 (63.04, 74.39)	55.87 (47.88, 63.57)	
Non-Hispanic Black	1,324 (22.56)	8.68 (6.43, 11.64)	10.91 (8.45, 13.98)	14.73 (11.71, 18.37)	
Other races	592 (10.09)	6.02 (4.72, 7.65)	7.02 (5.45, 8.99)	7.34 (5.75, 9.32)	
BMI (%)					0.4263
< 25	1,709 (29.11)	32.32 (28.97, 35.87)	30.92 (27.22, 34.88)	28.45 (25.19, 31.95)	
25–30	1,897 (32.32)	32.04 (28.25, 36.07)	32.61 (30.07, 35.25)	34.97 (31.58, 38.52)	
≥30	2,264 (38.57)	35.64 (32.38, 39.04)	36.47 (32.98, 40.11)	36.58 (33.80, 39.46)	
PIR (%)					< 0.0001
< 1.0	1,431 (24.38)	13.52 (11.32, 16.08)	14.00 (11.36, 17.14)	22.56 (19.69, 25.71)	
1.0–3.0	2,259 (38.48)	29.21 (26.02, 32.61)	33.49 (29.21, 38.06)	37.37 (34.38, 40.46)	
≥3.0	2,180 (37.14)	57.27 (52.99, 61.44)	52.51 (46.32, 58.62)	40.07 (35.71, 44.60)	
CVH (%)					0.0001
< 50	1,192 (20.31)	17.65 (15.16, 20.45)	14.28 (12.06, 16.84)	17.71 (15.41, 20.28)	
50–80	3,753 (63.94)	62.91 (59.81, 65.91)	62.78 (58.84, 66.55)	67.56 (64.42, 70.54)	
≥80	925 (15.76)	19.44 (15.92, 23.52)	22.94 (19.55, 26.72)	14.73 (12.35, 17.47)	
Education level (%)					< 0.0001
Less than high school	1,313 (22.37)	12.41 (10.06, 15.21)	11.50 (9.62, 13.70)	22.64 (19.88, 25.66)	
High school or GED	1,281 (21.82)	18.53 (14.94, 22.74)	20.99 (18.53, 23.68)	23.46 (20.70, 26.47)	
Above high school	3,276 (55.61)	69.06 (63.34, 74.25)	67.50 (63.88, 70.92)	53.90 (49.44, 58.29)	
Diabetes (%)					0.1526
Yes	5,157 (87.85)	10.16 (8.51, 12.09)	8.93 (7.44, 10.68)	7.80 (6.13, 9.87)	
No	713 (12.15)	89.84 (87.91, 91.49)	91.07 (89.32, 92.56)	92.20 (90.13, 93.87)	
Hypertension (%)					0.0001
Yes	4,142 (70.56)	30.25 (27.13, 33.58)	25.75 (22.74, 29.00)	21.27 (18.67, 24.12)	
No	1,728 (29.44)	69.75 (66.42, 72.87)	74.25 (71.00, 77.26)	78.73 (75.88, 81.33)	
Marital status (%)					< 0.0001
Married	2,914 (49.64)	60.65 (56.48, 64.67)	56.00 (51.85, 60.07)	44.69 (40.29, 49.17)	
Never married	1,331 (22.67)	17.60 (14.55, 21.13)	21.66 (18.00, 25.82)	24.86 (20.75, 29.48)	
Living with a partner	559 (9.52)	5.51 (4.46, 6.81)	7.96 (6.62, 9.55)	14.72 (12.43, 17.35)	
Other	1,066 (18.16)	16.24 (13.94, 18.83)	14.38 (12.22, 16.85)	15.72 (13.42, 18.34)	
Smoking status (%)					< 0.0001
Yes	3,277 (55.83)	45.09 (41.46, 48.78)	39.20 (35.39, 43.15)	49.26 (45.86, 52.66)	
No	2,593 (44.17)	54.91 (51.22, 58.54)	60.80 (56.85, 64.61)	50.74 (47.34, 54.14)	
Alcohol status (%)					0.6884
Yes	1,358 (23.13)	81.36 (78.42, 83.98)	82.91 (79.70, 85.70)	82.31 (79.59, 84.74)	
No	4,512 (76.87)	18.64 (16.02, 21.58)	17.09 (14.30, 20.30)	17.69 (15.26, 20.41)	
Oral microbiome diversity					< 0.0001
Observed ASVs	131.22 (44.37)	85.96 (84.82, 87.10)	127.54 (126.90, 128.18)	178.29 (176.37, 180.21)	< 0.0001
Faith's PD	14.60 (3.46)	11.09 (1.94)	14.47 (1.24)	18.24 (2.25)	< 0.0001
Shannon-Weiner index	4.62 (0.71)	4.06 (0.66)	4.63 (0.45)	5.16 (0.49)	< 0.0001
Simpson index	0.90 (0.07)	0.87 (0.09)	0.91 (0.05)	0.93 (0.04)	< 0.0001
Kidney stones (%)					0.0012
Yes	5,409 (92.15)	9.83 (8.03, 11.99)	7.86 (6.42, 9.58)	5.90 (4.76, 7.29)	
No	461 (7.85)	90.17 (88.01, 91.97)	92.14 (90.42, 93.58)	94.10 (92.71, 95.24)	

BMI, body mass index; PIR, poor income ratio; CVH, cardiovascular health; Faith's PD, Faith's Phylogenetic Diversity; Observed ASVs, observed amplicon sequence variants.

Oral microbiome diversity was described using observed ASVs. Values are weighted mean ± SD for continuous variables or weighted %for categorical variables.

### 3.2 Higher oral microbial alpha-diversity is associated with a lower risk of kidney stones

To explore whether alpha-diversity was associated with kidney stone risk, we analyzed different alpha-diversity indicators using multiple logistic regression (Table 2). When analyzed as continuous variables, the results showed that Observed ASVs (OR = 0.9973; 95% CI: 0.9949–0.9996), Faith's PD (OR = 0.9622; 95% CI: 0.9339–0.9914), Shannon-Weiner index (OR = 0.8571; 95% CI: 0.7479–0.9824), and Simpson Index (OR = 0.2425; 95% CI: 0.0645–0.9112), which were significantly and negatively associated with the risk of kidney stones (all *P* < 0.05). Taking observed ASVs as an example, our results showed that for each unit rise in observed ASVs, the risk of kidney stones decreased to 99.73% of the original risk. Further, we took alpha diversity as a categorical variable and analyzed it again. The results showed that after adjusting for all covariates, the higher alpha-diversity index still showed a significant negative association with kidney stone risk (all *P* < 0.05). Based on the reported ratios very close to 1, this may reflect the statistical significance associated with the large sample size. Therefore, we conducted a subject work characteristic curve (ROC) analysis and reported area under the curve (AUC) values, which indicated that the model had acceptable discriminatory power (Supplementary Figure S2). In addition, the results of the multicollinearity screen showed that the GVIF^(1/(2 × *Df*))^ values of all variables were between 1.04 and 1.55, which were much lower than the thresholds where severe covariance is usually considered to be present, suggesting that there was no significant multicollinearity problem among the variables (Supplementary Table S4). Therefore, we believe that the constructed model has good stability and interpretability.

**Table 2:** Multiple logistic regression analysis of the relationship between oral microbiome diversity and kidney stones.

**Oral microbiome diversity**	**OR (95%CI)**, ***P*** **value**		
	**Model 1**	**Model 2**	**Model 3**
Observed ASVs	0.9949 (0.9927, 0.9971) 0.000007	0.9974 (0.9951, 0.9997) 0.029459	0.9973 (0.9949, 0.9996) 0.024545
T1	Ref.	Ref.	Ref.
T2	0.8259 (0.6619, 1.307) 0.090600	0.9379 (0.7476, 1.1767) 0.579580	0.9477 (0.7535, 1.1919) 0.645791
T3	0.5871 (0.4614, 0.7472) 0.000015	0.7415 (0.5752, 0.9557) 0.020895	0.7305 (0.5644, 0.9454) 0.016998
Faith's PD	0.9345 (0.9086, 0.9611) 0.000002	0.9643 (0.9363, 0.9931) 0.015518	0.9622 (0.9339, 0.9914) 0.011518
T1	Ref.	Ref.	Ref.
T2	0.8098 (0.6482, 1.117) 0.063185	0.9298 (0.7401, 1.1683) 0.532277	0.9221 (0.7323, 1.1612) 0.490596
T3	0.6038 (0.4753, 0.7670) 0.000036	0.7641 (0.5940, 0.9829) 0.036213	0.7459 (0.5774, 0.9636) 0.024866
Shannon-Weiner index	0.7877 (0.6929, 0.8955) 0.000265	0.8584 (0.7500, 0.9824) 0.026602	0.8571 (0.7479, 0.9824) 0.026723
T1	Ref.	Ref.	Ref.
T2	0.6327 (0.5015, 0.7982) 0.000113	0.6732 (0.5318, 0.8522) 0.001005	0.6834 (0.5385, 0.8672) 0.001734
T3	0.6855 (0.5459, 0.8608) 0.001151	0.7846 (0.6200, 0.9929) 0.043494	0.7795 (0.6144, 0.9889) 0.040150
Simpson index	0.2374 (0.0669, 0.8426) 0.026078	0.2502 (0.0676, 0.9259) 0.037966	0.2425 (0.0645, 0.9112) 0.035933
T1	Ref.	Ref.	Ref.
T2	0.7233 (0.5721, 0.9143) 0.006754	0.7071 (0.5578, 0.8965) 0.004196	0.7203 (0.5670, 0.9150) 0.007190
T3	0.8356 (0.6663, 1.481) 0.120243	0.8640 (0.6858, 1.886) 0.214993	0.8560 (0.6780, 1.806) 0.190908

Model 1 was not adjusted for any variables; Model 2 was adjusted for age, gender, race, and ethnicity; Model 3 was adjusted for age, sex, race and ethnicity, BMI, PIR, CVH, education level, marital status, smoking status, alcohol status, hypertension, diabetes.

Faith's PD, Faith's Phylogenetic Diversity; Observed ASVs, observed amplicon sequence variants; BMI, body mass index; PIR, poor income ratio; CVH, cardiovascular health.

### 3.3 Dose-response association of high oral microbial alpha-diversity and low kidney stone risk

**Figure 2:**
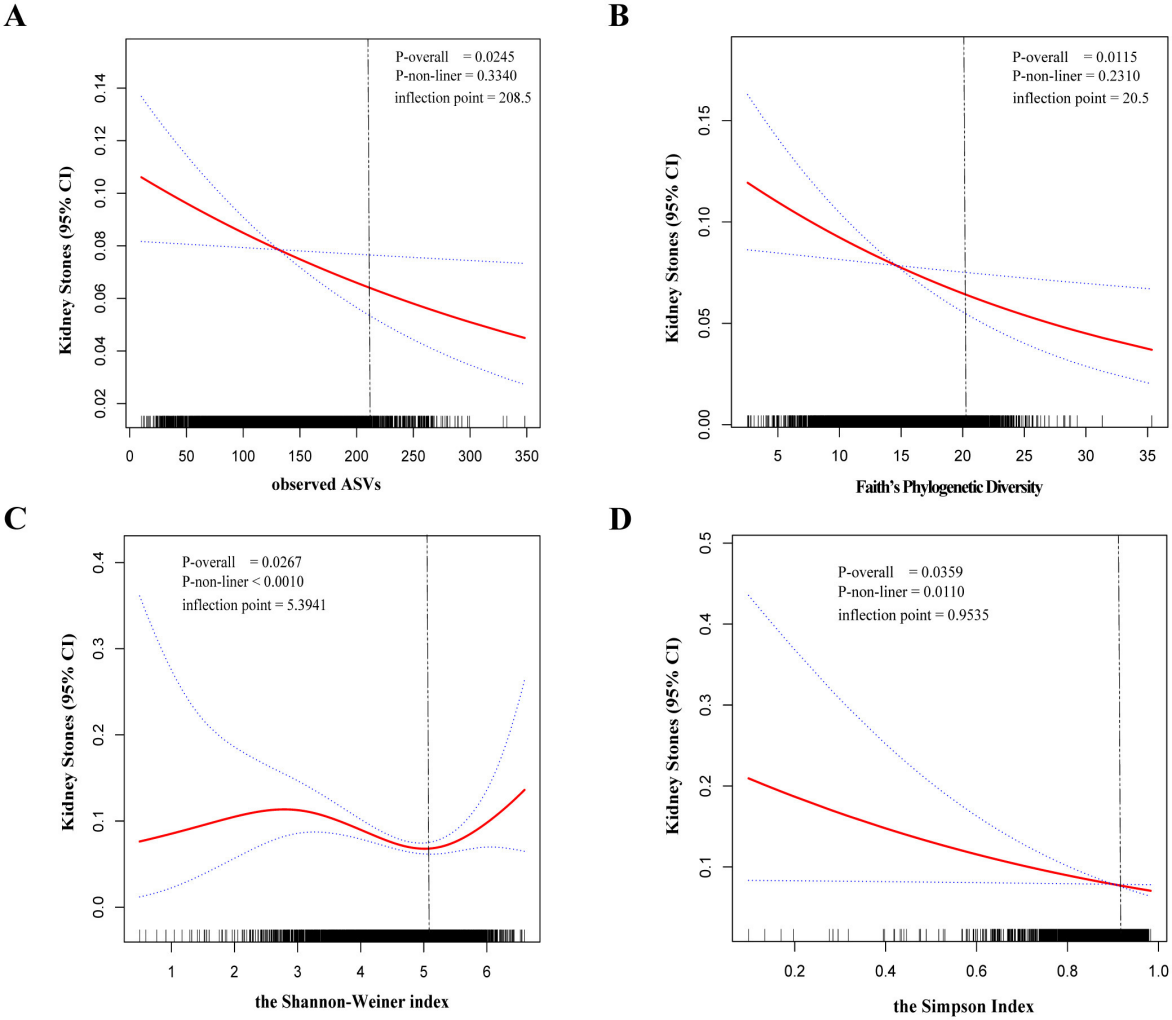
The dose-response relationship between the alpha-diversity index and kidney stones was assessed by restricted cubic spline regression. Observed ASVs **(A)**, Faith's Phylogenetic Diversity **(B)**, Shannon-Weiner index **(C)**, and Simpson Index **(D)**. The solid red line represents the smooth curve fit between variables. Blue bands represent the 95% confidence interval from the fit.

To further explore whether there was a dose-response relationship between alpha-diversity and kidney stone risk, we used restricted cubic spline to perform the analysis and subsequently quantified the results using saturation effect and threshold effect analyses (Table 3). For observed ASVs (Figure 2A) and Faith's PD (Figure 2B), the risk of kidney stones decreased with increasing alpha-diversity index. By quantifying the inflection points, we found a linear relationship between high Observed ASVs (*P*-non-liner = 0.3340) and Faith's PD (*P*-non-liner = 0.2310) and decreased risk of kidney stones. In contrast, the relationship between the Shannon-Weiner index (*P*-non-liner < 0.0010) and Simpson Index (*P*-non-liner = 0.0110) and decreased risk of kidney stones was non-linear. After measuring the Shannon-Weiner index inflection point, the risk of kidney stones first declined with the alpha-diversity index and then showed a significant increase. In addition, the risk of kidney stones decreased as the Simpson Index increased and then saturated. Therefore, the Shannon-Weiner index and kidney stone risk showed a specific “U” shaped association (Figure 2C), while the Simpson Index and kidney stone risk showed an inverse “J” shaped association (Figure 2D).

**Table 3:** Saturation and threshold effects between oral microbiome diversity and kidney stones.

	**OR (95%CI)**, ***P*** **value**			
**Oral microbiome diversity**	**Observed ASVs**	**Faith's PD**	**Shannon-Weiner index**	**Simpson index**
Inflection point	208.5	20.4831	5.3941	0.9535
< K slope 1	0.9968 (0.9942, 0.9993) 0.0140	0.9554 (0.9255, 0.9863) 0.0050	0.7679 (0.6634, 0.8889) 0.0004	0.1659 (0.0443, 0.6213) 0.0077
≥K slope 2	1.0052 (0.9900, 1.0206) 0.5058	1.0792 (0.9084, 1.2820) 0.3859	3.4920 (1.6424, 7.4244) 0.0012	inf. (26127.5798, inf.) 0.0090
Log-likelihood ratio test	0.334	0.231	< 0.001	0.011

Adjusted for age, sex, race and ethnicity, BMI, PIR, CVH, education level, marital status, smoking status, alcohol status, hypertension, and diabetes.

Faith's PD, Faith's Phylogenetic Diversity; Observed ASVs, observed amplicon sequence variants; BMI, body mass index; PIR, poor income ratio; CVH, cardiovascular health.

### 3.4 Subgroup analyses and interaction tests

Our results suggest that high alpha diversity is associated with a decreased risk of kidney stones, but sex-specific sensitivity analyses are necessary for different subgroups. Therefore, we used subgroup analyses and interaction tests to do so. By downscaling the data, we had previously identified observed ASVs as the main component. Therefore, we mainly showed subgroup analyses in which observed ASVs and kidney stone risk were negatively correlated (Table 4). Our results showed that oral microbiome diversity and kidney stone risk were negatively associated with significant differences in subgroups of people aged 40–60 years, male, obese, with CVH between 50 and 80, smokers, and without hypertension. Notably, we observed no significant interaction between observed ASVs and any of the subgroups (*P* for interaction > 0.05). In addition, we also analyzed and tested for interactions for different subgroups for Faith's PD (Supplementary Table S5), Shannon-Weiner index, and Simpson Index (Table 4). As well, which will not be repeated here.

**Table 4:** Subgroup analysis and interaction test for the association of oral microbiome diversity and kidney stones.

**Characteristics**	**ASVs**	***P* for interaction**	**Shannon-Weiner index**	***P* for interaction**	**Simpson index**	***P* for interaction**
	**OR (95% CI)** ***P*** **value**		**OR (95% CI)** ***P*** **value**		**OR (95% CI)** ***P*** **value**	
Age		0.5820		0.8947		0.9084
< 40	0.9969 (0.9923, 1.0015) 0.1830		0.8278 (0.6349, 1.0793) 0.1627		0.1576 (0.0140, 1.7727) 0.1346	
40–60	0.9952 (0.9915, 0.9989) 0.0119		0.8201 (0.6683, 1.0064) 0.0576		0.2754 (0.0421, 1.7999) 0.1782	
≥60	0.9985 (0.9942, 1.0029) 0.5046		0.8483 (0.6550, 1.0988) 0.2127		0.1801 (0.0099, 3.2889) 0.2474	
Gender		0.6126		0.2848		0.4615
Male	0.9967 (0.9935, 1.0000) 0.0476		0.9258 (0.7616, 1.1254) 0.4389		0.4625 (0.0533, 4.0119) 0.4842	
Female	0.9980 (0.9945, 1.0015) 0.2526		0.7985 (0.6606, 0.9652) 0.0200		0.1666 (0.0308, 0.9018) 0.0375	
Race and ethnicity		0.2370		0.5209		0.4844
Mexican American	0.9958 (0.9900, 1.0017) 0.1609		0.7928 (0.5533, 1.1359) 0.2057		0.0269 (0.0010, 0.7180) 0.0309	
Other Hispanic	0.9955 (0.9876, 1.0035) 0.2706		0.8701 (0.5260, 1.4394) 0.5880		0.8583 (0.0027, 271.3355) 0.9585	
Non-Hispanic White	0.9967 (0.9933, 1.0002) 0.0653		0.8281 (0.6888, 0.9955) 0.0447		0.3778 (0.0652, 2.1890) 0.2775	
Non-Hispanic Black	1.0009 (0.9953, 1.0066) 0.7484		0.9556 (0.6701, 1.3627) 0.8017		0.0933 (0.0022, 3.9568) 0.2148	
Other Races	0.9994 (0.9890, 1.0099) 0.9103		0.9289 (0.4933, 1.7494) 0.8194		0.2050 (0.0003, 140.8983) 0.6344	
BMI		0.8707		0.5534		0.6415
Normal weight	0.9962 (0.9907, 1.0017) 0.1773		0.7314 (0.5481, 0.9760) 0.0336		0.0912 (0.0068, 1.2241) 0.0707	
Overweight	0.9984 (0.9944, 1.0024) 0.4358		0.9252 (0.7281, 1.1757) 0.5249		0.3611 (0.0313, 4.1626) 0.4142	
Obese	0.9964 (0.9928, 0.9999) 0.0451		0.8457 (0.6887, 1.0384) 0.1095		0.2347 (0.0317, 1.7353) 0.1556	
CVH		0.3502		0.2203		0.2229
< 50	0.9982 (0.9940, 1.0023) 0.3879		0.8946 (0.6915, 1.1574) 0.3967		0.4024 (0.0202, 8.0020) 0.5507	
50–80	0.9967 (0.9937, 0.9998) 0.0375		0.8635 (0.7268, 1.0260) 0.0954		0.2699 (0.0543, 1.3423) 0.1095	
≥80	0.9927 (0.9826, 1.0028) 0.1553		0.5355 (0.3209, 0.8938) 0.0169		0.0065 (0.0001, 0.4007) 0.0166	
Smoking status		0.7894		0.3900		0.6248
Yes	0.9965 (0.9933, 0.9997) 0.0314		0.8018 (0.6665, 0.9645) 0.0191		0.1667 (0.0260, 1.0692) 0.0588	
No	0.9978 (0.9943, 1.0013) 0.2226		0.9036 (0.7381, 1.1063) 0.3264		0.3226 (0.0487, 2.1365) 0.2408	
Alcohol		0.7894		0.3074		0.1834
Yes	0.9974 (0.9947, 1.0001) 0.0590		0.8367 (0.7151, 0.9790) 0.0261		0.1595 (0.0349, 0.7287) 0.0179	
No	0.9982 (0.9931, 1.0033) 0.4800		0.9890 (0.7455, 1.3120) 0.9387		1.3312 (0.0757, 23.3986) 0.8449	
Hypertension		0.2386		0.2941		0.5090
Yes	0.9990 (0.9954, 1.0026) 0.5809		0.9359 (0.7571, 1.1568) 0.5399		0.4330 (0.0466, 4.0213) 0.4617	
No	0.9961 (0.9929, 0.9993) 0.0156		0.8073 (0.6757, 0.9645) 0.0183		0.1714 (0.0332, 0.8846) 0.0352	
Diabetes		0.3837		0.4407		0.2642
Yes	0.9952 (0.9899, 1.0005) 0.0731		0.7709 (0.5704, 1.0418) 0.0904		0.0468 (0.0021, 1.0621) 0.0546	
No	0.9978 (0.9951, 1.0004) 0.0959		0.8802 (0.7554, 1.0255) 0.1017		0.3428 (0.0766, 1.5337) 0.1614	

Adjusted for age, sex, race and ethnicity, BMI, PIR, CVH, education level, marital status, smoking status, alcohol status, hypertension, and diabetes.

Observed ASVs, observed amplicon sequence variants; BMI, body mass index; PIR, poor income ratio; CVH, cardiovascular health.

## 4 Discussion

This study investigated the association between oral microbiome diversity and kidney stones using data from a large, population-based survey in the United States. Surprisingly, alpha diversity was negatively associated with kidney stone risk. To ensure the reliability of the results, we conducted the corresponding sensitivity analysis assessment using restricted cubic spline curves and subgroup analysis.

Our study suggests that participants with high oral microbiome diversity were more likely to be young, male, less educated, not hypertensive, unmarried or living alone, and habitual smokers. These demographic and lifestyle factors are linked to a higher risk of kidney stone formation. It is well known that hypertensive patients have increased excretion of calcium and oxalate, which are key factors in stone formation (Poore et al., 2020). In addition, smoking may lead to oxidative stress and renal tubular damage, which may promote stone formation (Tsermpini et al., 2022; Caliri et al., 2021). Thus, the observed association between oral microbial diversity and these risk factors highlights the need for further studies in the future to investigate the potential mechanistic links between the oral microbiota and the pathogenesis of kidney stones. However, after controlling for these potential confounders in our multivariate regression model, the inverse relationship between oral microbiome diversity and the risk of kidney stones remained statistically significant. This suggests that oral microbiome diversity is not solely driven by these baseline characteristics in renal stone pathogenesis and may play an independent protective role rather than being associated with traditional risk factors. The observed paradox therefore highlights the complexity of host-microbiome interactions and emphasizes the need for further mechanistic studies to explore potential causal pathways linking oral microbial ecology to kidney stone formation.

Our results suggest that both the Shannon-Weiner index and Simpson Index are protective factors against the pathogenesis of kidney stones (OR < 1). This suggests that higher oral microbial diversity may contribute to reducing the risk of kidney stone formation. Although the two indices differ in their mathematical expression, they both capture fundamental aspects of species richness and evenness. Interestingly, the heat map shows a positive correlation between the two. This suggests that they are internally consistent and that individuals with higher overall microbial diversity may have more favorable microbial environments, thereby mitigating stone pathogenesis. Previous studies have demonstrated that dysbiosis can elevate oxidative stress and chronic low-grade inflammation, both of which are established risk factors for kidney stone formation (Mostafavi Abdolmaleky and Zhou, 2024). Furthermore, certain microbial metabolites may influence urine composition, including oxalate and citrate levels, thereby directly impacting the risk of stone formation (Gao et al., 2022). Thus, the observed association between higher oral microbial diversity and lower risk of kidney stones may reflect a more balanced microbial ecosystem that helps to maintain host metabolic and immune homeostasis. These findings warrant further investigation of the functional role of specific microbial taxa on diversity-related protective effects.

The model constructed in this study to assess the association between oral microbial diversity and the risk of kidney stones showed an AUC of about 0.696 on ROC analysis, indicating that the model had an acceptable discriminatory power (Zhang et al., 2015). Despite the “highly accurate” criterion, this predictive performance is still informative in epidemiologic studies, suggesting a potential role of oral microecology in kidney stone formation, considering that the occurrence of kidney stones may be influenced by a combination of environmental, genetic, and lifestyle factors.

Previous research has examined the link between the oral microbiome and mortality, establishing a negative association, which provides a basis for our current study (Yang et al., 2024). Existing research suggests that the oral microbiota plays a crucial role in overall body health (Santacroce et al., 2023). Oral microbiota diversity is often considered an indicator of good health and can impact systemic health by enhancing the immune system, modulating inflammation, and participating in metabolic processes (Wang et al., 2024). Imbalances in the oral microbiota may impact kidney health by promoting systemic inflammation, thereby increasing the risk of chronic kidney disease. This imbalance may be transmitted via the bloodstream, triggering a systemic immune response that subsequently affects kidney function and stone formation (Mizutani et al., 2022).

Caution should be exercised in interpreting the negative correlation association between the oral microbiome and kidney stones because the oral microbiome has specific characteristics in particular diseases. First, the oral microbiota interacts with the gut microbiota via the mouth-gut axis (Tan et al., 2023). Pathogenic bacteria in the oral cavity may reach the gut through swallowing, thereby influencing gut microbiota diversity (Wu et al., 2024). The gut microbiota is closely linked to urinary metabolites (e.g., calcium, oxalic acid, and uric acid), and alterations in these metabolites may indirectly influence kidney stone formation (Al et al., 2023). A study confirmed that gut microbiota diversity influences the absorption of calcium and oxalic acid, subsequently affecting urinary oxalic acid levels, a key factor in kidney stone formation (Yuan et al., 2023). Second, the oral microbiota influences kidney health through immune regulation, and oral microbial diversity may promote immune tolerance, preventing excessive immune responses (Liu et al., 2022). Studies have shown that imbalances in the oral microbiota are linked to elevated systemic inflammation, which may promote kidney stone formation (Knauf et al., 2019). Another study confirmed that pathogenic bacteria in the oral cavity may enter the bloodstream, triggering an immune response that affects kidney immune function and metabolism (Guo et al., 2022). In addition, several studies have revealed the role of the oral microbiota in mineral metabolism in the body. The oral microbiota may influence systemic metabolism via metabolites, such as short-chain fatty acids, which are critical for stone formation. Short-chain fatty acids have been shown to reduce the risk of stones by inhibiting oxalic acid absorption in the gut or promoting its excretion (Blaak et al., 2020).

Emerging evidence indicates that certain oral microbial taxa may actively contribute to systemic health and potentially affect the development of kidney stones (Xu et al., 2025). For instance, genera such as Streptococcus, Prevotella, and Veillonella are frequently detected in individuals with greater oral microbial diversity and have been associated with the regulation of inflammatory pathways (Li et al., 2022; Chen et al., 2023). Some of these microbes are known to produce short-chain fatty acids (SCFAs), which exert systemic anti-inflammatory effects and may help preserve renal homeostasis (Fusco et al., 2023). Others may alter the local pH or produce ammonia, potentially disrupting the systemic acid-base balance (Weiner and Verlander, 2019). Additionally, changes in the oral microbiota may affect nitric oxide metabolism or urea recycling, thereby indirectly influencing urinary composition, including oxalate, citrate, and calcium levels (Bryan et al., 2022; Rosier et al., 2024). These mechanisms offer potential explanations for the observed protective association between greater oral microbial diversity and reduced kidney stone risk.

Consequently, we hypothesized that higher microbial diversity is generally associated with better health, as diverse microbial communities can better maintain ecological balance and inhibit pathogenic bacterial growth. In the context of kidney stones, oral microbiota diversity may reflect stronger systemic immune responses and metabolic stability, thereby reducing the risk of stone formation. The oral microbiota may indirectly influence renal metabolic processes by affecting the gut microbiota, immune system, and hepatic and renal metabolism, thus contributing to kidney stone formation or prevention.

To the best of our knowledge, this is the first study to explore the relationship between oral microbiome diversity and kidney stones in a large, population-based sample. The strengths of this study include, among others, our inclusion of a nationally representative sample of U.S. adults, as well as innovative sensitivity analyses for different indicators and subgroups and dose-response relationships. However, we have to recognize some limitations. First, our study was an observational design and cannot demonstrate a causal and temporal difference between oral microbiome diversity and kidney stones. Second, the consideration of covariates may be incomplete. Third, although NHANES collects information about kidney stones through professionally trained personnel, it does not, however, detail stone composition or frequency of stone episodes, which could introduce potential bias. Therefore, further prospective designs and causality studies are needed to confirm our findings.

## 5 Conclusions

In this study, we found an inverse relationship between oral microbiome diversity and kidney stone risk observed in alpha diversity. This reveals the complexity of host-microbiome interactions, and further mechanistic studies are necessary to elucidate these complex roles in the future.

## Data Availability

Publicly available datasets were analyzed in this study. This data can be found here: https://www.cdc.gov/nchs/nhanes/.
