# Supplementary material for: Association between oral microbiome diversity and kidney stones: a cross-sectional study
Source: Front Microbiol. 2025 May 29;16:1600961. doi: 10.3389/fmicb.2025.1600961 (PMC12159058; doi:10.3389/fmicb.2025.1600961)

Supplementary Material

**Supplementary Table 1.** Definition and scoring approach for the American Heart Association’s Life’s Essential 8 score.

| Domain | CVH Metric | Measurement | Quantification and Scoring of CVH Metric |
| --- | --- | --- | --- |
| Health Behaviors | 1.Diet | Healthy Eating Index-2015 diet score percentile | Quantiles of DASH-style diet adherence  Scoring (Population):  Points Quantile  100 ≥95th percentile (top/ideal diet)  80 75th – 94th percentile  50 50th – 74th percentile  25 25th – 49th percentile  0 1st – 24th percentile (bottom/least ideal quartile) |
|  | 2.Physical activity | Self-reported minutes of moderate or vigorous physical activity per week | Metric: Minutes of moderate (or greater) intensity activity per week  Scoring:  Points Minutes  100 ≥150  90 120 – 149  80 90 – 119  60 60 – 89  40 30 – 59  20 1 – 29  0 0 |
|  | 3.Nicotine exposure | Self-reported use of cigarettes or inhaled nicotine- delivery system | Metric: Combustible tobacco use and/or inhaled NDS use; or secondhand smoke exposure  Scoring:  Points Status  100 Never smoker  75 Former smoker, quit ≥5 yrs  50 Former smoker, quit 1 - <5 yrs  25 Former smoker, quit <1 year, or currently using inhaled NDS  0 Current smoker  Subtract 20 points (unless score is 0) for living with active indoor smoker in home |
|  | 4.Sleep health | Self-reported average hours of sleep per night | Metric: Average hours of sleep per night  Scoring:  Points Level  100 7 – <9  90 9 – <10  70 6 – <7  40 5 – <6 or ≥10  20 4 – <5  0 <4 |

| Domain | CVH Metric | Measurement | Quantification and Scoring of CVH Metric |
| --- | --- | --- | --- |
| Health Factors | 1.Body mass index | Body weight (kg) divided by height squared (m2) | Metric: Body mass index (kg/m2)  Scoring: Points Level 100 <25  70 25.0 – 29.9  30 30.0 – 34.9  15 35.0 – 39.9  0 ≥40.0 |
|  | 2.Blood lipids | Plasma total and HDL-cholesterol with calculation of non-HDL-cholesterol | Metric: Non-HDL-cholesterol (mg/dL)  Scoring:  Points Level  100 <130  60 130 – 159  40 160 – 189  20 190 – 219  0 ≥220  If drug-treated level, subtract 20 points |
|  | 3.Blood glucose | Fasting blood glucose or casual hemoglobin A1c | Metric: Fasting blood glucose (mg/dL) or Hemoglobin A1c (%)  Scoring:  Points Level  100 No history of diabetes and FBG <100 (or HbA1c < 5.7)  60 No diabetes and FBG 100 – 125 (or HbA1c 5.7-6.4) (Pre-diabetes)  40 Diabetes with HbA1c <7.0  30 Diabetes with HbA1c 7.0 – 7.9  20 Diabetes with HbA1c 8.0 – 8.9  10 Diabetes with Hb A1c 9.0 – 9.9  0 Diabetes with HbA1c ≥10.0 |
|  | 4.Blood pressure | Appropriately measured systolic and diastolic blood pressure | Metric: Systolic and diastolic blood pressure (mm Hg)  Scoring:  Points Level  100 <120/<80 (Optimal)  75 120-129/<80 (Elevated)  50 130-139 or 80-89 (Stage I HTN)  25 140-159 or 90-99  0 ≥160 or ≥100  Subtract 20 points if treated level |

# Supplementary Table 2. KNots used in RCS curves and corresponding actual values

| Characteristics | The actual value corresponding to the quantile | | |
| --- | --- | --- | --- |
|  | 10% | 50% | 90% |
| observed_ASVs | 78.50 | 128.60 | 190.05 |
| Faiths_Phylogenetic_Diversity | 10.45 | 14.50 | 18.99 |
| the_ShannonWeiner_index | 3.76 | 4.67 | 5.45 |
| the_Simpson_Index | 0.83 | 0.92 | 0.95 |

**Supplementary Table 3.** Pearson's correlation coefficients for different α-diversity metrics.

|  | observed_ASVs | Faiths_Phylogenetic_Diversity | the_ShannonWeiner_index | the_Simpson_Index |
| --- | --- | --- | --- | --- |
| observed_ASVs | 1 | 0.9597491 | 0.73408566 | 0.41167664 |
| Faiths_Phylogenetic_Diversity | 0.9597491 | 1 | 0.70404311 | 0.39349484 |
| the_ShannonWeiner_index | 0.73408566 | 0.70404311 | 1 | 0.84542472 |
| the_Simpson_Index | 0.41167664 | 0.39349484 | 0.84542472 | 1 |

**Supplementary Table 4.** Screening for covariance between covariates

| VariableName | GVIF | Df | GVIF^(1/(2*Df)) |
| --- | --- | --- | --- |
| AGE | 1.616525 | 1 | 1.271426 |
| GENDER | 1.13425 | 1 | 1.065012 |
| RACE | 1.330204 | 4 | 1.03631 |
| PIR | 1.447955 | 1 | 1.20331 |
| EDUCATION | 1.415734 | 2 | 1.090801 |
| MARITAL | 1.381648 | 3 | 1.055357 |
| ALCOHOL | 1.216231 | 1 | 1.102829 |
| SMOKE | 1.337207 | 1 | 1.156377 |
| HEI2015_ALL | 1.380875 | 1 | 1.175107 |
| BMI | 1.470201 | 1 | 1.212518 |
| DIABETES | 1.24253 | 1 | 1.114688 |
| HYPERTENSION | 1.348761 | 1 | 1.161362 |
| CVH | 2.397487 | 1 | 1.548382 |

**Supplementary Table 5.** Subgroup analysis and interaction test for the association of oral microbiome diversity (Faith’s Phylogenetic Diversity) and kidney stones.

| Characteristics | OR(95% CI) P value | P for interaction |
| --- | --- | --- |
| Age |  | 0.3184 |
| ＜40 | 0.9443 (0.8883, 1.0038) 0.0660 |  |
| 40-60 | 0.9440 (0.9018, 0.9883) 0.0137 |  |
| ≥60 | 0.9821 (0.9306, 1.0365) 0.5114 |  |
| Gender |  | 0.5872 |
| Male | 0.9552 (0.9172, 0.9949) 0.0272 |  |
| Female | 0.9710 (0.9296, 1.0143) 0.1859 |  |
| Race and ethnicity |  | 0.1349 |
| Mexican American | 0.9433 (0.8758, 1.0159) 0.1230 |  |
| Other Hispanic | 0.9065 (0.8178, 1.0048) 0.0617 |  |
| Non-Hispanic White | 0.9570 (0.9166, 0.9992) 0.0460 |  |
| Non-Hispanic Black | 1.0178 (0.9480, 1.0928) 0.6262 |  |
| Other Races | 0.9853 (0.8606, 1.1281) 0.8303 |  |
| BMI |  | 0.7852 |
| Normal weight | 0.9583 (0.8937, 1.0275) 0.2314 |  |
| Overweight | 0.9684 (0.9211, 1.0182) 0.2097 |  |
| Obese | 0.9522 (0.9108, 0.9955) 0.0308 |  |
| CVH |  | 0.2949 |
| < 50 | 0.9761 (0.9268, 1.0279) 0.3590 |  |
| 50-80 | 0.9532 (0.9170, 0.9909) 0.0153 |  |
| ≥ 80 | 0.9113 (0.8020, 1.0355) 0.1541 |  |
| Smoking status |  | 0.9206 |
| Yes | 0.9591 (0.9216, 0.9981) 0.0401 |  |
| No | 0.9620 (0.9198, 1.0061) 0.0903 |  |
| Alcohol |  | 0.7663 |
| Yes | 0.9635 (0.9309, 0.9973) 0.0346 |  |
| No | 0.9739 (0.9158, 1.0356) 0.3985 |  |
| Hypertension |  | 0.1575 |
| Yes | 0.9869 (0.9432, 1.0326) 0.5691 |  |
| No | 0.9450 (0.9082, 0.9833) 0.0053 |  |
| Diabetes |  | 0.6330 |
| Yes | 0.9483 (0.8882, 1.0124) 0.1117 |  |
| No | 0.9653 (0.9336, 0.9980) 0.0377 |  |

Note: Adjusted for age, sex, race and ethnicity, BMI, PIR, CVH, education level, marital status, smoking status, alcohol status, hypertension, diabetes. BMI, body mass index; PIR, poor income ratio; CVH, cardiovascular health.

**Supplementary Figure 1.** The pairwise Pearson correlation coefficient between different alpha diversity metrics.


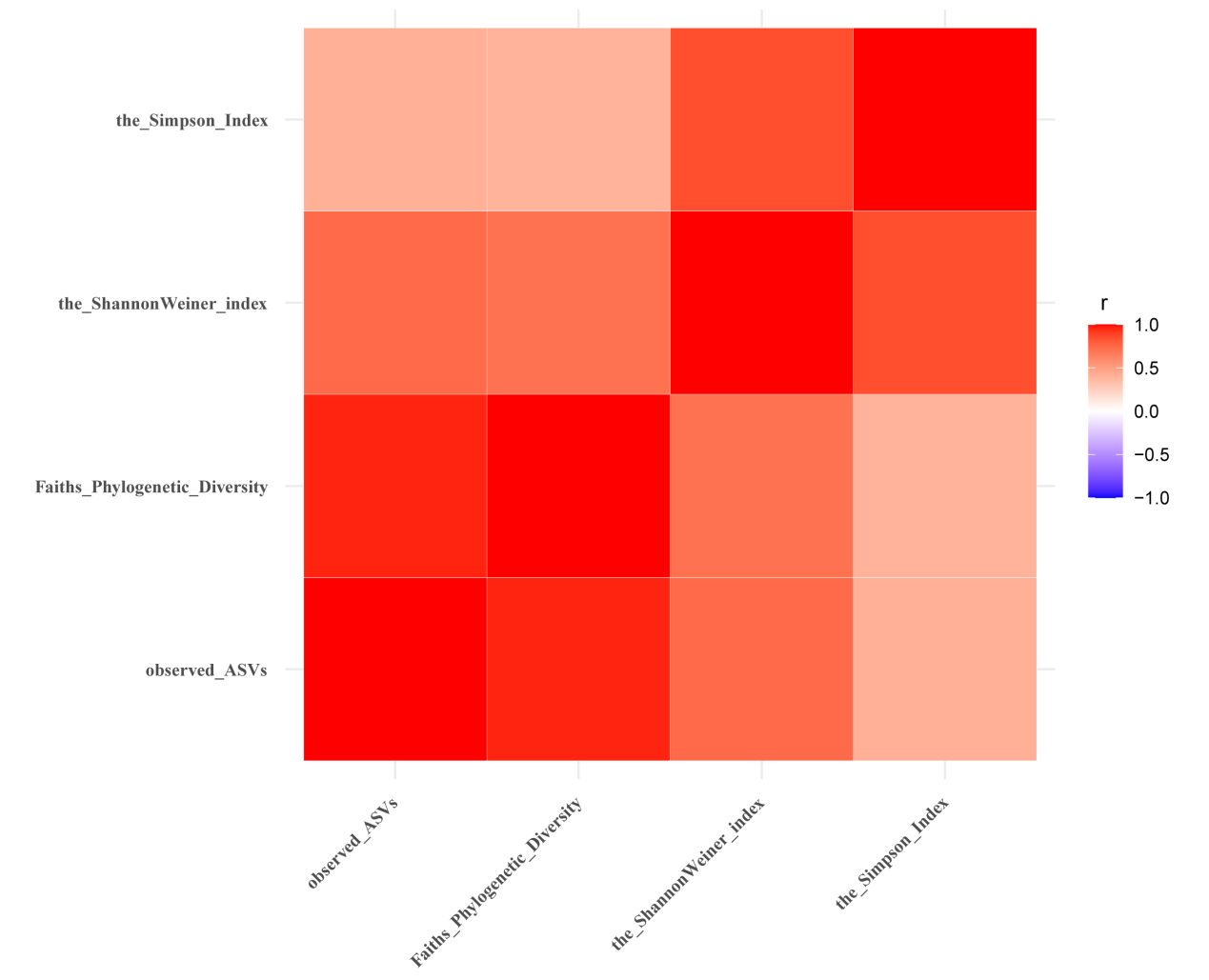


**Supplementary Figure 2.** Work characterization curve analysis of the oral microbial diversity model for subjects with kidney stones.


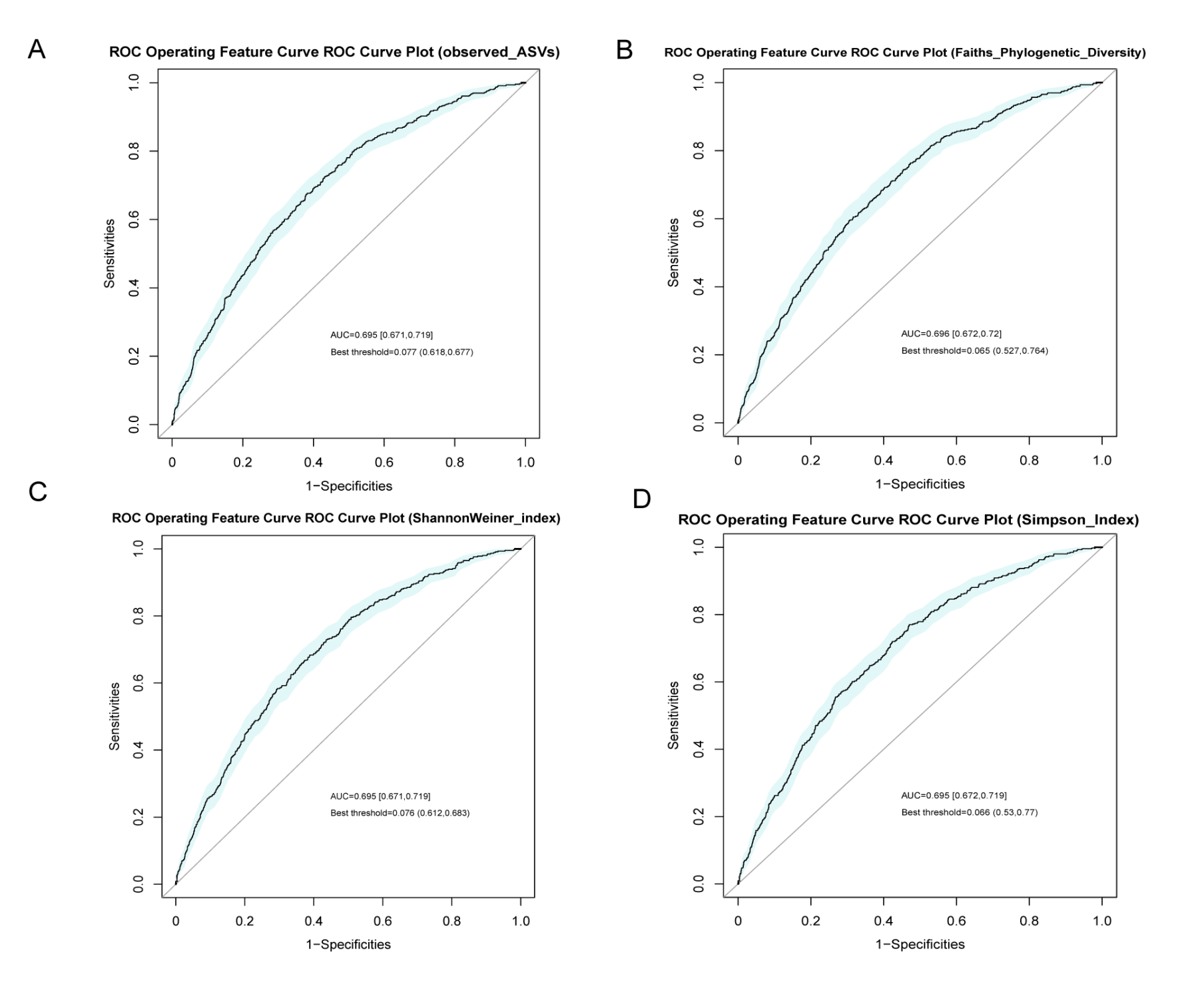

Supplement: Supplementary file 1 [file Data_Sheet_1.docx]
